# Plant Flavoprotein Photoreceptors

**DOI:** 10.1093/pcp/pcu196

**Published:** 2014-12-15

**Authors:** John M. Christie, Lisa Blackwood, Jan Petersen, Stuart Sullivan

**Affiliations:** Institute of Molecular Cell and Systems Biology, College of Medical, Veterinary and Life Sciences, University of Glasgow, Glasgow G12 8QQ, UK

**Keywords:** Blue light, Chromophore, Cryptochrome, Flavin, Phototropin, Zeitlupe family

## Abstract

Plants depend on the surrounding light environment to direct their growth. Blue light (300–500 nm) in particular acts to promote a wide variety of photomorphogenic responses including seedling establishment, phototropism and circadian clock regulation. Several different classes of flavin-based photoreceptors have been identified that mediate the effects of blue light in the dicotyledonous genetic model *Arabidopsis thaliana*. These include the cryptochromes, the phototropins and members of the Zeitlupe family. In this review, we discuss recent advances, which contribute to our understanding of how these photosensory systems are activated by blue light and how they initiate signaling to regulate diverse aspects of plant development.

## Introduction

Light is recognized ubiquitously throughout nature as a source of energy. For many organisms, including plants and algae, light is an important environmental stimulus that directs their development, morphogenesis and physiology. This is achieved by specialized photoreceptors that detect and respond to changes in light intensity, quality, direction and duration. These light-responsive proteins typically contain a prosthetic cofactor or chromophore that enables them to perceive and respond to specific wavelengths of light. To date, five photosensory systems have been identified in higher plants. The phytochromes (phys) respond largely to red (600–700 nm) and far-red (700–750 nm) regions of the solar spectrum (Chen and Chory 2011), whereas UV Resistance locus 8 (UVR8) monitors ultraviolet B (UV-B) wavelengths (280–315 nm) to regulate both developmental and UV-protective processes (Jenkins 2014). Plant responses to blue light (390–500 nm) are extensive and are mediated by three different classes of photoreceptor: the cryptochromes (crys) (Chaves et al. 2011), phototropins (phots) (Christie 2007) and members of the Zeitlupe family (ztl, fkf1 and lkp2) (Suetsugu and Wada 2013).

**Fig. 1:**
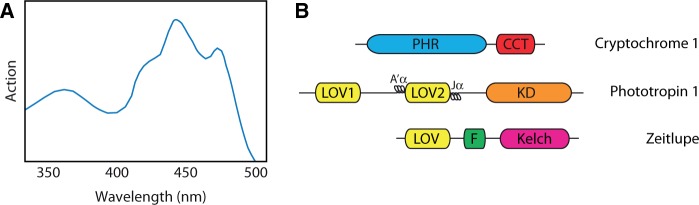
Action and domain structure of plant flavoprotein blue-light receptors. (A) Stylized representation of a typical action spectrum for phototropism. (B) Domain structures of cryptochrome 1, phototropin 1 and zeitlupe: PHR, photolyase homology region; CCT, cryptochrome C-terminus; LOV, light, oxygen or voltage sensing; KD, kinase domain; F, F-box; Kelch, kelch repeats.

Genetic analyses in the model flowering plant *Arabidopsis thaliana* have been instrumental in defining the molecular basis of plant blue-light receptors and their mechanism of action. However, action spectra for a number of blue-light-initiated responses were described prior to the advent of Arabidopsis genetics and provided important information regarding the photochemical properties of the photoreceptors involved. For instance, the action spectrum for phototropism resembles the absorption spectrum of a flavoprotein; it shows maximal activity between 400 and 500 nm and reveals a degree of fine structure with a major peak at 450 nm and subsidiary shoulders at 430 and 470 nm (Fig. 1A). An additional broad, less effective peak is typically observed in the UV-A region of the spectrum at 380 nm. Such properties are common to all plant blue-light receptors as each class binds oxidized flavin as a light-absorbing chromophore (Conrad et al. 2014).

Here, we summarize how light activates these different classes of flavoprotein photoreceptors to regulate a variety of blue light responses in Arabidopsis. Throughout this review we will adopt the nomenclature first introduced for Arabidopsis phys (Quail et al. 1994) and phots (Briggs et al. 2001) where the holoprotein with its chromophore photoreceptor is designated in lower case (cry, phot and ztl) and in upper case when the apoprotein without its chromophore is described (CRY, PHOT and ZTL). By convention, we also use upper case italics for the genes encoding the photoreceptor apoproteins (*CRY*, *PHOT* and *ZTL*) and lower case italics to refer to mutant alleles (*cry*, *phot* and *ztl*).

## Cryptochromes

Cryptochromes were the first plant blue-light receptors to be characterized at the molecular level (Ahmad and Cashmore 1993). They are major regulators of plant photomorphogenic development (also referred to as de-etiolation) and function to control the photoperiodic control of flowering in addition to entrainment of the circadian clock (Liu et al. 2011). Cryptochromes exhibit significant homology to photolyases, which are blue-light-activated enzymes found in both prokaryotes and eukaryotes that catalyze the light-dependent repair of damaged DNA produced from exposure to UV-B irradiation (Ahmad and Cashmore 1993). Cryptochromes together with the photolyases comprise a superfamily of proteins that can be subdivided into five groups: cyclobutane pyrimidine dimer (CPD) photolyases, (6–4) pyrimidine–pyrimidone adduct [(6–4) photoproduct] photolyases, cry-DASH (*Drosophila*–Arabidopsis–Synechocystis–Human) proteins, animal cryptochromes and plant cryptochromes (Chaves et al. 2011).

Three cryptochromes have been identified in Arabidopsis (cry1–cry3). Arabidopsis cry1 and cry2 are localized predominantly in the nucleus (Cashmore et al. 1999, Kleiner et al. 1999). They do not exhibit DNA repair activity but function to control various aspects of plant growth and development; cry1 has a major role in regulating seedling de-etiolation under blue light, whereas cry2 is involved regulating flowering in response to day-length (Liu et al. 2011). Cry2 differs from cry1 in that it undergoes blue-light-dependent ubiquitination and degradation in the nucleus (Zuo et al. 2012). Hence, cry2 functions preferentially under low light conditions or where light is limiting. Cry3 is a cry-DASH protein that localizes to mitochondria and chloroplasts (Kleine et al. 2003) and is reported to repair UV-induced lesions in single-stranded DNA (Selby and Sancar 2006) as well as in loop structures of double-stranded DNA (Pokorny et al. 2008).

### Cryptochrome structure and light sensing

Arabidopsis cryptochromes, like photolyases, bind two chromophores: FAD and a pterin derivative 5,10-methenyltetrahydrofolate (MTHF). FAD is bound non-covalently within the photolyase homology region (PHR) and functions as the primary light sensor (Fig. 1B), whereas the role of MTHF, as is the case for photolyases, is to harvest and transfer additional light energy to the FAD chromophore from the near UV region (370–390 nm) (Hoang et al. 2008). In addition to the PHR domain (∼500 amino acids), cry1 and cry2 also contain a distinctive cryptochrome C-terminus (CCT) that is absent from cry3, cry-DASH proteins and other photolyases (Fig. 1B). While photosensing is mediated by the PHR domain, the CCT (∼100–200 amino acids) is important for cryptochrome signaling (Yang et al. 2000, Yu et al. 2009).

**Fig. 2:**
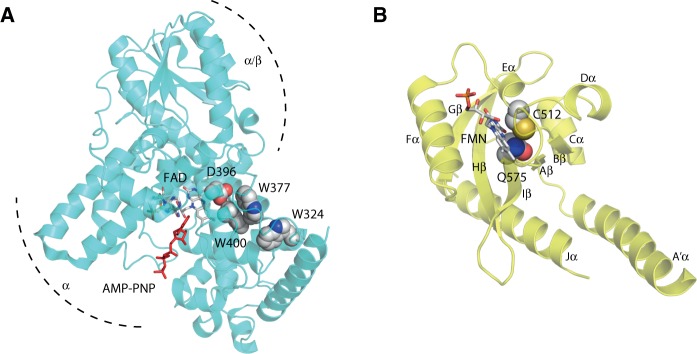
Structural features of the chromophore-binding regions of cry1 and phot1. (A) Structure of the photolyase homology region of Arabidopsis cry1 in the dark state with the non-hydrolyzable ATP analog adenylyl-imidodiphosphate (AMP-PNP) bound (Brautigam et al. 2004). N-terminal α/β and C-terminal α domains are indicated along with the FAD chromophore. Residues proposed to be important for photoactivation are shown as spheres. (B) Structure of the LOV2 domain of Arabidopsis phot1 in the dark state (Halavaty and Moffat 2013). Positions of the FMN chromophore, the A′α- and Jα-helix are indicated, as are residues important for photoactivation.

Light sensing by the PHR domain is proposed to promote conformational changes within the CCT that are necessary for interacting with downstream signaling components. The crystal structure of the PHR domain of Arabidopsis cry1 was solved a decade ago (Brautigam et al. 2004). Structural information for the CCT is still lacking at present. The PHR domain of cry1 shows strong structural similarity to photolyases (Fig. 2A). The PHR fold comprises an N-terminal α/β domain (residues 13–139) and a C-terminal α domain (residues 217–495). A connecting region of 77 amino acids links these two domains together. The FAD chromophore is buried within a hydrophobic U-shaped pocket inside the α domain. The binding site for MTHF has been solved at the structural level for cry3 (Klar et al. 2007), but not for cry1 or cry2, since this accessory chromophore is typically lost upon their purification from heterologous expression systems. In cry3, MTHF is bound at a distance of 15.2 Å from the FAD chromophore. The MTHF-binding region is highly conserved in DASH-type cryptochromes. However, a structural comparison between cry3 and cry1 suggests some differences in MTHF binding within this region (Klar et al. 2007).

**Fig. 3:**
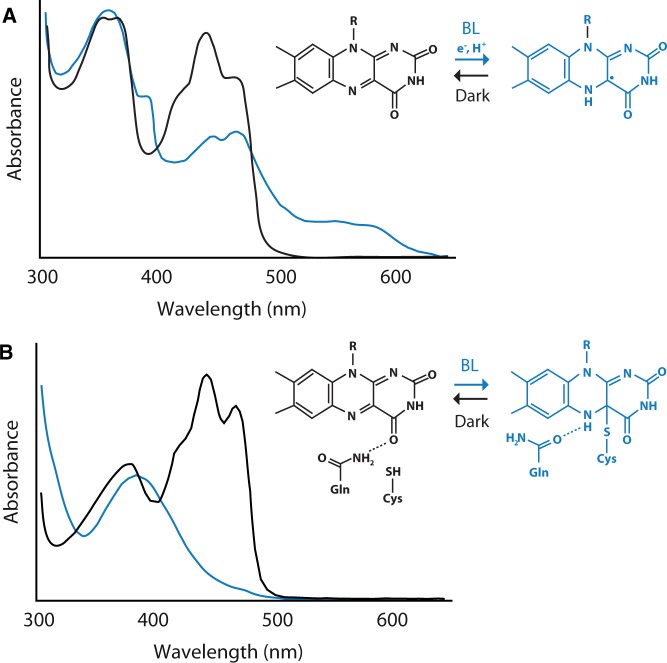
Photochemical reactivity of the PHR domain and the LOV domain. (A) The graph illustrates the light-induced absorbance changes observed for the PHR domain of Arabidopsis cry2 (Banerjee et al. 2007). The black line represents the absorption spectrum of the dark state. The blue line represents the absorption spectrum following blue light irradiation. Blue light results in the formation of a semi-reduced neutral FAD radical (FADH·), which is generated by photoreduction and protonation of the FAD chromophore (inset). (B) The graph illustrates the light-induced absorbance changes observed for Arabidopsis phot1 LOV2 (Jones et al. 2007). The black line represents the absorption spectrum of the dark state. The blue line represents the absorption spectrum following blue light irradiation. Blue light induces formation of a covalent adduct between the FMN chromophore and a conserved cysteine residue within LOV2 (inset). Adduct formation results in side chain rotation of a nearby glutamine residue and altered hydrogen bonding with the FMN chromophore.

Spectroscopic analysis of recombinant cry1 shows that it binds fully oxidized FAD in the inactive or resting state, which strongly absorbs blue light (λ_max_ ∼450 nm). Irradiation of the PHR domain results in the formation of a semi-reduced (semiquinone) neutral FAD radical (FADH·). FADH· is generated by photoreduction and subsequent protonation of the FAD chromophore (Fig. 3A). Light-driven generation of FADH· occurs within microseconds (Langenbacher et al. 2009) and is considered to represent the signaling state that triggers photoreceptor activation. In the absence of light, FADH· is reportedly unstable and can revert back to FAD within minutes (Chaves et al. 2011). Inhibitory effects of green light have been observed on cryptochrome function presumably by antagonizing FADH· formation, which absorbs in this spectral region (Fig. 3A). Recombinant cryptochromes from Arabidopsis therefore undergo a reversible photocycle between FAD and FADH· states.

Photoreduction of the FAD cofactor is proposed to involve a conserved flavin-reducing triad of tryptophan residues (Chaves et al. 2011) within the α domain (W400, W377 and W324). W400 is the predicted electron donor proximal to the flavin whereby W324 is exposed to the protein surface (Fig. 2A). This photoreduction mechanism is still under investigation, but is reported to involve transfer of electrons between the FAD and the tryptophan cascade as well as protonation of the flavin by the putative aspartic acid proton donor D396 (Cailliez et al. 2014). Mutation of the residues within the tryptophan triad to alanine impairs flavin photoreduction in recombinant cry2, providing support for this reaction mechanism, at least in vitro (Li et al. 2011). Similar mutations have also been used effectively to impact the photoreduction of cry1 (Zeugner et al. 2005, Bouly et al. 2007).

While mutation of the tryptophan triad impairs flavin photoreduction in vitro, structure–function studies in Arabidopsis are conflicted regarding its biological role in cryptochrome photoactivation. Arabidopsis mutants lacking cry1 and cry2 are impaired in de-etiolation responses and exhibit a long hypocotyl and small, unopened cotyledons when grown in continuous blue light (Liu et al. 2011). Triad mutations W400F and W324F failed to restore cry1 function when expressed in cry-deficient mutants, consistent with a role in photoreduction and light sensing (Zeugner et al. 2005). Comparable mutations have also been reported to impact the blue-light-dependent degradation of cry2 (Li et al. 2011). However, mutations in the tryptophan triad of cry2 have been shown to elicit different functional effects compared with those observed for cry1. Mutation of the surface-exposed tryptophan in cry2 (W321) weakened its activity, while side chain replacements of the remaining tryptophans (W397 and W374) resulted in constitutive cry2 activity (Li et al. 2011). It therefore seems likely that mutation of W397 and W394 causes structural alterations within the CRY2 apoprotein such that it adopts an activate conformation in the absence of light. Hence, the biological role of the tryptophan triad in cryptochrome photoactivation is still under debate (Liu et al. 2010). Indeed, recent studies indicate that alternative electron transport pathways, independent of the tryptophan triad, are also involved in flavin photoreduction (Engelhard et al. 2014).

### Cryptochrome activation

**Fig. 4:**
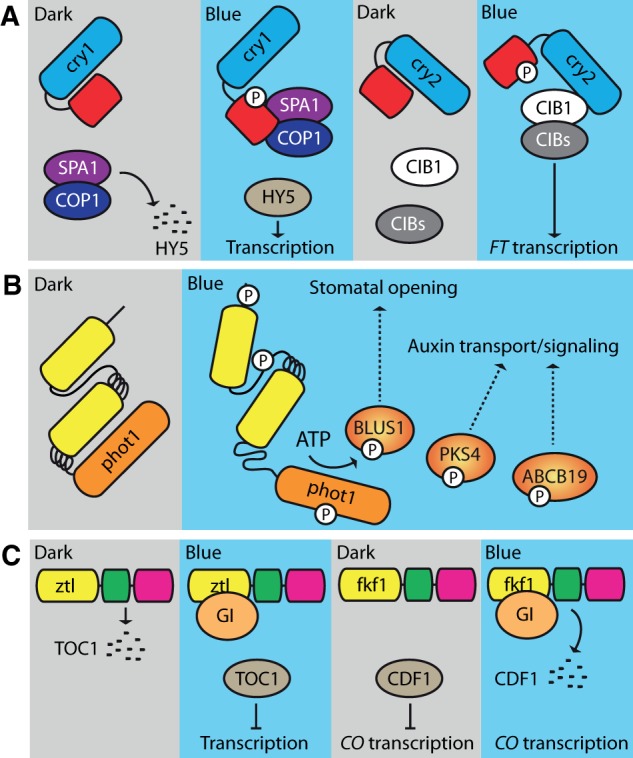
Primary signaling events associated with plant blue-light receptors. (A) Cryptochrome photoactivation and indirect regulation of transcription by cry1 (left). In darkness, HY5 is degraded by the action of the COP1–SPA1 complex. In blue light, cry1 alters its structure to bind to and sequester COP1–SPA1, resulting in the accumulation of HY5 and the promotion of gene expression. Direct regulation of transcription by cry2 (right). Upon photoactivation, cry2 binds to CIB1 and other CIB proteins via its PHR domain to promote flowering by inducing gene expression including *FT*. (B) Light-induced autophosphorylation and substrate phosphorylation by phot1. In the dark state, phot1 kinase activity is inhibited. Blue light drives a conformational change in the protein that results in autophosphorylation within the kinase domain, the linker region between the LOV domains and sequences upstream of LOV1. Concomitantly, phot1 can phosphorylate substrate targets including BLUS1, PKS4 and ABCB19. (C) Photoactivation of ztl and stabilization of TOC1 (left). In darkness, ztl functions to degrade TOC1. GI binding to ztl in blue light results in TOC1 accumulation and the transcriptional repression of circadian genes by inhibiting SCF^ztl^ activity. Blue-light-induced binding of GI to fkf1 (right) results in the degradation of the transcriptional repressor CDF1 to promote *CO* expression and flowering under long days.

Despite the lack of structural information on the CCT, its role in initiating crytochrome signaling is well established (Chaves et al. 2011, Liu et al. 2011). The PHR domain and the CCT are proposed to form a closed or inactive conformation in darkness (Fig. 4A). Blue light sensing by the PHR domain would then trigger a conformational change in the CCT to produce an open or active conformation that initiates signaling. Consistent with this mode of action, light-induced conformational changes associated with the CCT of cry1 have been reported using a variety of techniques, including limited proteolysis (Partch et al. 2005), pulsed laser-induced transient grating (Kondoh et al. 2011) and Fourier transform infrared (FTIR) difference spectroscopy (Kottke et al. 2006). Furthermore, overexpression of the CCT in Arabidopsis confers constitutive cryptochrome signaling (Yang et al. 2000, Yu et al. 2009), suggesting that this region can adopt an active conformation in the absence of the PHR domain. Indeed, mutation of residues G380 and L470 within Arabidopsis cry1 appears to impact interdomain association between the PHR domain and the CCT, leading to constitutive activation in the absence of light (Exner et al. 2010, Gu et al. 2012).

The mechanism(s) by which the FAD to FADH· transition within the PHR domain is propagated to bring about the proposed conformational change within the CCT is still not fully understood. However, recent studies suggest that a contributing factor might involve the ability of cryptochrome to bind ATP. The PHR domain of Arabidopsis cry1 was co-crystallized with a non-hydrolyzable ATP analog (AMP-PNP), which binds close to the flavin cofactor (Fig. 2A). ATP binding is reported to both increase the yield and prolong the lifetime of the FADH· signaling state (Immeln et al. 2007, Burney et al. 2009, Cailliez et al. 2014, Muller et al. 2014). Specifically, Muller et al. (2014) suggest that ATP binding increases the p*K*_a_ of the potential aspartic acid proton donor D396 to facilitate its protonation (D396H) under physiological pH conditions and ensure that the FAD remains in close contact with W400 to promote electron transfer (Cailliez et al. 2014). The deprotonation of D396 (D396^−^) that occurs upon light-driven FADH· formation would be expected to introduce strong electrostatic changes within its hydrophobic environment that could trigger conformational changes necessary for cryptochrome activation.

Phosphorylation of the CCT has also been shown to correlate closely with the photoactivation and biological function of plant cryptochromes (Liu et al. 2011). Both Arabidopsis cry1 and cry2 become rapidly phosphorylated in etiolated seedlings upon exposure to blue light (Shalitin et al. 2002, Shalitin et al. 2003). Phosphorylation on multiple serine residues could be expected to promote electrostatic repulsion of the CCT from the PHR domain upon exposure to light (Liu et al. 2011). Several different protein kinases are likely to be involved in CCT phosphorylation. In the case of cry2, CCT phosphorylation by casein kinase 1 appears to induce ubiquitination and subsequent degradation of the protein (Tan et al. 2013). The observation that Arabidopsis cry1 purified from insect cells is phosphorylated upon photoexcitation, combined with its ability to bind ATP, has led to the proposal that cry1 itself exhibits autophosphorylation activity (Bouly et al. 2003, Shalitin et al. 2003). However, given that cry1 shows no homology to protein kinases, phosphorylation of the CCT by a co-purifying kinase following its exposure upon irradiation cannot be excluded entirely from these studies.

### Cryptochrome signaling

Nuclear-localized cry1 and cry2 largely mediate their effects on plant development through changes in gene expression (Chaves et al. 2011, Liu et al. 2011). Approximately 5–25% of the gene expression changes that occur during seedling de-etiolation under blue light can be attributed to the action of cry1 and cry2 in Arabidopsis (Folta and Spalding 2001, Ma et al. 2001, Ohgishi et al. 2004). Two mechanisms of transcriptional control have been elucidated for plant cryptochromes. The first mechanism involves modulating the abundance of positive regulators, including the bZIP transcription factor Long Hypocotyl 5 (HY5), by suppressing their degradation in response to light (Osterlund et al. 2000). In etiolated seedlings, HY5 is targeted for proteolysis by the Constitutive Photomorphogenic 1 (COP1)/Suppressor of phyA 1 (SPA1) complex. COP1–SPA1 acts as a substrate receptor for the CUL4–DDB1 E3 ubiquitin ligase complex, which is responsible for degrading proteins that promote photomorphogenic development (Lau and Deng 2012). Upon photoactivation, cry1 and cry2 can bind SPA1 and suppress the action of the COP1–SPA1 complex (Fig. 4A). This COP1–SPA1–cry1 interaction leads to an accumulation of HY5 and other factors, which, in turn, regulate the transcription of genes required for de-etiolation. Expression of constitutively active forms of plant cryptochromes, including the truncated CCT region, can impair this mode of regulation and permanently sequester the COP1–SPA1 complex from degrading HY5, giving rise to a light-grown or constitutive photomorphogenic (*cop*) phenotype (short hypocotyl and opened cotyledons) when seedlings are grown in darkness (Yang et al. 2000, Yu et al. 2009). Similarly, light-driven interactions between COP1–SPA1 and cry2 have been shown to suppress proteolysis of the zinc finger transcriptional regulator Constans (CO), a key regulator of flowering in response to long days (Zuo et al. 2011). Light-dependent degradation of cry2 may also involve the action of SPA1 in concert with phyA (Weidler et al. 2012).

In addition to controlling the abundance of transcription factors such as HY5 through the COP1–SPA1 pathway, cry2 has been reported to interact directly with and modulate the activity of transcription factors associated with the photoperiodic control of flowering (Fig. 4A). Upon photoactivation, cry2 binds the basic helix–loop–helix (bHLH) transcription factor cry-interacting bHLH1 (CIB1) via its PHR domain (Kennedy et al. 2010) to promote expression of *Flowering Locus T* (*FT*) (Liu et al. 2008), a mobile transcriptional regulator of floral initiation. Other CIB family members, including CIB2 and CIB5, bind to cry2 and function redundantly in regulating cry2-dependent flowering (Liu et al. 2013). However, CIB1 does not appear to bind the *FT* promoter directly (Fig. 4A), but instead heterodimerizes with other CIB homologs to fulfill this function (Liu et al. 2013).

Although predominantly localized to the nucleus, evidence suggests that cry1 can also influence signaling events at the plasma membrane. Blue light induces a transient depolarization of the plasma membrane in Arabidopsis (Spalding 2000) that is linked to hypocotyl growth inhibition (Cho and Spalding 1996). Nuclear-localized cry1 is sufficient to promote this response (Wu and Spalding 2007). Moreover, membrane depolarization is rapid (occurring within 30 s), implying that the nucleus–plasma membrane communication mechanism involved is too rapid to include changes in gene expression. Yet, the signaling events that couple photoactivation of cry1 in the nucleus to plasma membrane depolarization remain largely unexplored. As discussed below, a separate class of flavoprotein photoreceptor known as the phototropins largely mediates blue light sensing at the plasma membrane.

## Phototropins

Phototropin blue-light receptors were named after their role in mediating higher plant phototropism (Christie et al. 1999). The absorption properties of these photoreceptors correspond closely to the action spectra for this response (Figs. 1A, 3B). Phototropins are light-activated serine/threonine kinases that undergo autophosphorylation in response to blue light irradiation. Arabidopsis contains two phototropins (phot1 and phot2), which regulate a range of photoresponses that serve to optimize photosynthetic efficiency and promote growth particularly under weak light conditions (Takemiya et al. 2005). Both phot1 and phot2 are predominantly localized to the plasma membrane (Sakamoto and Briggs 2002, Kong et al. 2006), but are not integral membrane proteins. Instead, they are attached to the intracellular side of the plasma membrane and can be released by treatment with non-ionic detergents (Knieb et al. 2004, Kong et al. 2013a). Their mode of membrane attachment is still not clearly defined, but may involve peptide sequences residing within the extreme C-terminal region of the protein (Kong et al. 2013b). Recent reports suggest that phot1 in addition to phot2 can localize to the outer membrane of the chloroplast, consistent with their role in regulating chloroplast photorelocation movements (Kong et al. 2013b).

Blue light irradiation has been shown to elicit different impacts on the subcellular localization of phot1 and phot2. A fraction of phot1 re-localizes from the plasma membrane to the cytosol upon irradiation (Sakamoto and Briggs 2002, Han et al. 2008, Wan et al. 2008, Kaiserli et al. 2009), whereas phot2 is targeted to the Golgi apparatus (Kong et al. 2006, Kong et al. 2007, Aihara et al. 2008, Aggarwal et al. 2014). Translocation from the plasma membrane is inhibited when the kinase activities of phot1 (Kaiserli et al. 2009) and phot2 (Kong et al. 2006, Kong et al. 2007, Aggarwal et al. 2014) are impaired. Although the biological significance of these translocation processes is still not known, internalization of phot1 has been linked to modulating receptor signaling, at least for phototropism (Wan et al. 2008).

Genetic analysis in Arabidopsis has shown that phot1 and phot2 overlap in function to regulate hypocotyl and root phototropism (Sakai et al. 2001), chloroplast accumulation movement (Kagawa et al. 2001), stomatal opening (Kinoshita et al. 2001), leaf positioning and leaf flattening (Sakamoto and Briggs 2002, Inoue et al. 2008b). In the case of hypocotyl phototropism, phot2 functions predominantly at high light intensities, whereas phot1 acts over a broad range of fluence rates (Sakai et al. 2001). Phot2, on the other hand, is solely responsible for eliciting chloroplast and nuclear avoidance movements in response to high intensity blue light (Kagawa et al. 2001, Higa et al. 2014). An additional role for phot2 in regulating resistance protein-mediated viral defense has been reported (Jeong et al. 2010). Phot1-specific responses have also been identified and include the rapid inhibition of hypocotyl growth upon transfer of etiolated seedlings to light (Folta et al. 2003), transcript destabilization (Folta and Kaufman 2003) and, more recently, the suppression of lateral root growth (Moni et al. 2014).

### Phototropin structure and light sensing

Phototropins comprise a serine/threonine kinase domain at their C-terminus and two specialized light, oxygen or voltage sensing (LOV) domains, designated LOV1 and LOV2, at their N-terminus (Fig. 1B). LOV1 and LOV2 function as blue light sensors and bind oxidized FMN non-covalently as chromophore (Christie et al. 1999). The structural and biophysical properties of the LOV domain have been studied extensively and provide a valuable framework to understand how the phototropin light switch operates (Christie et al. 2012). LOV domains (∼110 amino acids) form a subset of the Per-ARNT-Sim (PAS) superfamily and consist primarily of five antiparallel β-sheets (Aβ, Bβ, Gβ, Hβ and Iβ) and several α-helices (Cα, Dα, Eα and Fα) (Freddolino et al. 2013). The FMN chromophore is bound tightly inside these structural elements, which comprise the LOV/PAS core (Fig. 2B).

Spectroscopic analyses of recombinant LOV domains obtained from a variety of phototropin proteins show that they bind oxidized FMN in the inactive state, absorbing strongly in the blue region of the spectrum (λ_max_ ∼450 nm). Like the PHR domain of plant cryptochromes, purified LOV domains are photochemically active in solution (Fig. 3B). Irradiation of the LOV domain results in the formation of a covalent bond between the C(4a) carbon of the FMN and the sulfur atom of a nearby, conserved cysteine residue (Salomon et al. 2000, Swartz et al. 2001). FMN-cysteinyl adduct formation occurs within microseconds of illumination and produces a spectral species that no longer absorbs blue light (Fig. 3B). FMN-cysteinyl adduct formation represents the active signaling state that leads to photoreceptor activation. This photochemical reaction is fully reversible in darkness. Thermal decay of the covalent adduct back to the dark state occurs within tens to thousands of seconds depending on the LOV domain (Christie et al. 2012).

Recombinant protein fragments of Arabidopsis phot1 and phot2 containing both LOV domains (LOV1 + 2) exhibit more complex photochemistry than either domain alone. For instance, adduct-decay kinetics for LOV1 + 2 proteins are noticeably slower compared with individual LOV domains, taking on the order of tens of minutes to recover (Kasahara et al. 2002). This slower rate of adduct decay is proposed to enhance receptor photosensitivity by prolonging the lifetime of the photoproduct (Christie et al. 2002). Conversely, modest acceleration of the LOV photocycle can be achieved, at least in vitro, by simultaneous or subsequent irradiation with UV-A light (Kennis et al. 2004), which antagonizes FMN-cysteinyl adduct formation which absorbs in this region (Δ_max_ ∼390 nm; Fig. 2B). Moreover, specific amino acid changes within the FMN-binding pocket have been identified that significantly accelerate or slow down the rate of adduct decay of the LOV photocycle (Christie et al. 2007, Zoltowski et al. 2009, Circolone et al. 2012, Kawano et al. 2013, Zayner and Sosnick 2014). These findings therefore open up new possibilities to fine-tune photoproduct life time and modulate photoreceptor activity.

Mutation of the conserved cysteine involved in adduct formation (Fig. 2B) abolishes the photochemical reactivity of the LOV domain (Salomon et al. 2000, Swartz et al. 2001) and has been used to functionally dissect the roles of LOV1 and LOV2 in regulating phot1 activity. The C512A mutation that block the photochemical reactivity of LOV2 fails to restore phot1 function when expressed in *phot1*-deficient mutants of Arabidopsis (Christie et al. 2002). These and other structure–function studies highlight the importance of LOV2 in regulating light-dependent autophosphorylation, which is essential for phototropin function (Cho et al. 2007, Jones et al. 2007). Yet, similar mutational analyses on phot2 suggest that LOV1 can still have a residual role in light sensing when LOV2 is photochemically inactivated, at least for phototropism (Cho et al. 2007, Suetsugu et al. 2013). Further structure–function analyses in Arabidopsis provide additional evidence for a photoactive role for the LOV1 domain of phot1 in arresting chloroplast accumulation movement at high light intensities (Kaiserli et al. 2009). Such a role could serve to promote an efficient transition from chloroplast accumulation to avoidance movement under excess light. Additional work is now needed to clarify the roles of LOV1 and LOV2 and whether these differ between phot1 and phot2. Although the functional significance of LOV1 still remains unclear, its presence is proposed to modulate LOV2 photoreactivity (Christie et al. 2002, Sullivan et al. 2008, Okajima et al. 2014) and play a role in receptor dimerization (Nakasako et al. 2008).

### Phototropin activation

There is presently no crystal structure for the entire phototropin molecule. However, recent FTIR difference spectroscopy (Pfeifer et al. 2010) and small-angle X-ray scattering (SAXS) combined with low resolution molecular modeling (Okajima et al. 2014) have provided insights into the domain architecture of the photoreceptor, as well as the global structural changes that occur upon photoexcitation. SAXS analyses suggest that LOV1, LOV2 and the kinase domain (KD) are arranged sequentially within the molecule, with LOV1 and the KD being positioned on either side of LOV2 (Fig. 4B). Such an arrangement is consistent with a model whereby LOV2 functions closely with the KD to act as the main regulator of phototropin activity (Christie 2007, Tokutomi et al. 2008). In a mechanism comparable with that depicted for cryptochrome activation (Fig. 4A), the N-terminal photosensory region of phototropin (comprising LOV1 and LOV2) is hypothesized to form a closed or inactive conformation with the C-terminal KD in darkness, in which LOV2 together with adjacent regulatory sequences act to repress phototropin kinase activity. Blue light sensing by LOV2 results in a conformational change that causes the N-terminus to move and alleviate this repression (Fig. 4B), which leads to an opening of the catalytic cleft between the N- and C-terminal lobes of the KD to promote ATP binding and initiate receptor autophosphorylation (Pfeifer et al. 2010).

The mechanism by which FMN-cysteinyl adduct formation in LOV2 is propagated to bring about a global light-driven conformational change initially involves a conserved glutamine residue within LOV2 which hydrogen-bonds with the O4 oxygen of the FMN chromophore in darkness (Fig. 3B). FMN-cysteinyl adduct formation results in side chain rotation of this glutamine to alter its hydrogen bonding with the FMN chromophore temporarily (Crosson and Moffat 2001). Q575 resides within the β-scaffold of Arabidopsis phot1 LOV2, which acts as a docking site for a conserved α-helix (Jα) located outside the LOV core (Fig. 2B). Side chain rotation of this glutamine is proposed to invoke structural alterations within the β-sheet surface that lead to displacement and unfolding of the Jα-helix flanking the C-terminus of the LOV2 core (Harper et al. 2003, Freddolino et al. 2013). Mutation of this glutamine in the LOV2 domain of Arabidopsis phot1 attenuates light-activated disordering of the Jα-helix (Nash et al. 2008) and consequently impacts receptor autophosphorylation (Jones et al. 2007). Jα-helix displacement therefore represents an integral component of the phototropin light switch. Indeed, artificial disruption of the Jα-helix from the LOV2 core through targeted mutagenesis uncouples this mode of regulation and leads to phot1 activation in the absence of light (Harper et al. 2004, Jones et al. 2007, Kaiserli et al. 2009).

Recent crystallographic studies (Halavaty and Moffat 2013) have shown that sequences flanking the N-terminus of LOV2 also comprise an α-helix (Fig. 2B). Several lines of evidence suggest that this α-helix (A′α) plays a key role in regulating LOV2 activity in concert with Jα (Zayner et al. 2012, Freddolino et al. 2013, Takeda et al. 2013). Mutations within this region appear to disrupt the repressive action of LOV2 on phototropin kinase activity (Aihara et al. 2012). Consequently, mutations in A′α have adverse effects on phototropin signaling (Sharma et al. 2014). Together, these findings highlight the importance of A′α together with the Jα-helix in activating the LOV2 photoswitch.

### Phototropin autophosphorylation and signaling

A primary consequence of phototropin activation is receptor autophoshorylation (Christie et al. 1998). Phosphorylation occurs predominantly on multiple serine residues, and several studies have been successful in mapping the sites involved. So far at least 21 phosphorylation sites have been identified in Arabidopsis phot1 (Salomon et al. 2003, Inoue et al. 2008a, Sullivan et al. 2008, Boex-Fontvieille et al. 2014, Deng et al. 2014) and 29 in Arabidopsis phot2 (Inoue et al. 2011, Boex-Fontvieille et al. 2014). These sites are listed in Table 1 for convenience. Structure–function studies carried out to date indicate that autophosphorylation at two conserved serine residues within the kinase activation loop or T-loop (S849 and S851 in phot1; S761 and S763 in phot2) is essential for receptor signaling in Arabidopsis (Inoue et al. 2008a, Inoue et al. 2011). The majority of the remaining phosphorylation sites reside within either the N-terminal region upstream of LOV1 or the linker sequence between LOV1 and LOV2 (Fig. 4B). Although the biological significance of these upstream phosphorylation sites remains largely unexplored, the occurrence of some of these phosphoserines is fluence rate dependent (Salomon et al. 2003), suggesting that they may have different biochemical consequences.

**Table 1:** List of the in vivo phosphorylation sites identified for Arabidopsis phot1 and phot2

	phot1	phot2
N-terminus	S12, S58, S92, S141, T144, S165, S166, S170, S185	S9, S22, S30, T34, S37, T38, S39, S53, S54, T67, S88, S105, S106, S111, S112, S114, S121
LOV1–LOV2	S350, T353, T360, S364, S376, S406, S409, S410, S442, S450	S284, S289, S291, T294, S300, S301, T302, T303, T305, S319, T328, S364
Kinase domain	S849 and/or S851	
C-terminus	T993	

The relative positions within the domain structure of the protein are indicated on the left.

At least for phot1, phosphorylation at S350, S376 and S410 located within the linker region between LOV1 and LOV2 is required to bind members of the non-epsilon group of Arabidopsis 14-3-3 regulatory proteins (Inoue et al. 2008a, Sullivan et al. 2009). Mutation of the 14-3-3-binding site does not appear to affect the functionality of phot1 in Arabidopsis (Inoue et al. 2008a). In contrast, binding of 14-3-3λ to phot2 is reported to involve S747 within the KD. Mutation of this site impairs phot2-mediated stomatal opening, but fails to impact phot2-meidated phototropism, as well as leaf positioning and flattening (Tseng et al. 2012). These findings suggest that 14-3-3 binding to phot2 is an important signaling step for stomatal opening. Whether an equivalent serine mediates a similar function for phot1 awaits further investigation.

The mechanisms underlying blue-light-induced stomatal opening have been extensively studied and therefore represent one of the best characterized phototropin signaling pathways (Inoue et al. 2010). The plasma membrane H^+^-ATPase, together with inward-rectifying K^+^ channels, is a key component of this pathway. Phototropin-mediated activation of the H^+^-ATPase induces hyperpolarization of the guard cell plasma membrane, which allows K^+^ uptake through inward-rectifying K^+^ channels (Shimazaki et al. 2007). Accumulation of K^+^ induces the swelling of the guard cells, which promotes stomatal opening. Activation of the guard cell H^+^-ATPase involves phosphorylation and subsequent 14-3-3 binding at its C-terminus (Kinoshita and Shimazaki 1999). The kinase responsible for phosphorylating the H^+^-ATPase has not been identified. However, recent work has shown that phototropin activation of the H^+^-ATPase is initiated by the guard cell-specific kinase known as Blue Light Signaling 1 (BLUS1). BLUS1 is directly phosphorylated by both phot1 and phot2 (Fig. 4B) at S348 located within its C-terminus (Takemiya et al. 2013a). Phosphorylation of S348 and BLUS1 kinase activity are both essential for activation of the H^+^-ATPase. As a result, Arabidopsis mutants lacking BLUS1 show no blue-light-induced stomatal opening, but exhibit normal phototropism, chloroplast relocation movement and leaf flattening (Takemiya et al. 2013a). Phototropin-mediated phosphorylation of BLUS1 therefore represents a key primary step in the signal cascade leading to phosphorylation and activation of the H^+^-ATPase. The signaling events coupling BLUS1 phosphorylation to activation of the H^+^-ATPase are still not well defined, but are known to involve protein phosphatase 1 activity (Takemiya et al. 2013b).

Two other phosphorylation targets have been reported for Arabidopsis phot1 besides BLUS1. The auxin efflux carrier ABCB19 is a member of the ATP-binding cassette B family of transporter proteins (Spalding 2013). Phot1 can phosphorylate ABCB19 in vitro and has been shown to inhibit ABCB19 transporter activity when co-expressed in HeLa cells (Christie et al. 2011). Nevertheless, this process is not required to establish the lateral auxin gradient that drives phototropic growth. Instead, phot1-mediated inhibition of ABCB19 appears to function in enhancing phototropic responsiveness (Noh et al. 2003, Nagashima et al. 2008). In addition to ABCB19, members of the Phytochrome Kinase Substrate (PKS) family have also been shown to play a role in phototropism. PKS4 is rapidly phosphorylated by phot1 in response to blue light and is proposed to impact phototropism positively or negatively depending on its phosphorylation status (Demarsy et al. 2012, Kami et al. 2014). The biochemical function of PKS proteins is presently unknown, but they probably impact auxin signaling or transport (Kami et al. 2014). For a more detailed description of the signaling events associated with phototropism, readers are directed to several recently published reviews for more information regarding phototropic signaling (Sakai and Haga 2012, Christie and Murphy 2013, Goyal et al. 2013, Hohm et al. 2013, Liscum et al. 2014), as well as comprehensive overviews covering the mechanisms underlying phototropin-mediated chloroplast photorelocation movement (Wada 2013, Kong and Wada 2014).

The search for additional phototropin substrate targets is undoubtedly ongoing. In addition to autophosphorylation, Arabidopsis phot1 becomes ubiquitinated following blue light irradiation (Roberts et al. 2011). Mass spectrometry analysis has identified K526 within the Fα-helix of the LOV2 core as a site of ubiquitination (Deng et al. 2014). Ubiquitination requires the phot1-interacting protein Non-Phototropic Hypocotyl 3 (NPH3) (Roberts et al. 2011), which appears to function as part of a Cullin 3-based E3 ubiquitin ligase complex that regulates protein degradation and subcellular trafficking (Roberts et al. 2011, Wan et al. 2012). Further elucidation of the biochemical action of NPH3 and related proteins such as Root Phototropism Protein 2 (RPT2; Sakai and Haga 2012) is now essential to understanding their role in phototropin signaling, particularly phototropism.

## Zeitlupe and Related Blue-Light Receptors

Several other LOV domain-containing photoreceptors exist in Arabidopsis besides the phototropins. Members of the ztl family play important roles in controlling the degradation and stability of components associated with circadian clock regulation and the photoperiodic control of flowering. Ztl members localize to the cytosol or the nucleus (Takase et al. 2011) and comprise three members: Zeitlupe (ztl), Flavin-binding kelch repeat F-box 1 (fkf1) and LOV kelch protein 2 (lkp2) (Ito et al. 2012, Suetsugu and Wada 2013). Genetic analysis in Arabidopsis indicates that ztl, fkf1 and lkp2 partially overlap in function (Fornara et al. 2009, Baudry et al. 2010, Takase et al. 2011). Arabidopsis mutants lacking ztl are primarily impaired in circadian clock function (Somers et al. 2000), whereas *fkf1* mutants mostly show alterations in flowering time (Imaizumi et al. 2003). While mutants lacking lkp2 show minimal alterations in circadian regulation and flowering (Baudry et al. 2010), overexpression of *LPK2* in Arabidopsis compromises both these processes (Schultz et al. 2001).

Members of the ztl family contain a LOV domain at their N-terminus followed by an F-box and six kelch repeats at their C-terminus (Fig. 1B). F-boxes are associated with Skp Cullin F-box (SCF)-type E3 ubiquitin ligases, which target proteins for degradation via the ubiquitin–proteosome system (Ito et al. 2012), whereas kelch repeats serve to mediate protein–protein interactions and heterodimerization between lkp2 and the other two family members (Takase et al. 2011). In principle, the primary protein architecture of the ztl family is similar to that of cryptochrome and phototropin where the photosensory module is located upstream from a C-terminal effector region. Phototropin is, however, the only blue-light receptor identified to date that contains two LOV domains.

Recombinant LOV domains derived from ztl, fkf1 and lkp2 bind oxidized FMN as chromophore and show photochemical reactivity analogous to that of phototropin LOV domains, whereby blue light irradiation drives the formation of an FMN-cysteinyl adduct within the protein (Imaizumi et al. 2003). The photocycle of the LOV domain of fkf1 differs substantially from that of phototropin LOV domains in that adduct decay is extremely slow, occurring in the order of days (Zikihara et al. 2006). These slow photocycle properties have been attributed to the presence of a nine amino acid insertion between Eα and Fα that is absent from phototropin LOV domains (Zikihara et al. 2006). This failure to photocycle rapidly does not appear to impact the ability of fkf1 to respond to day-length since both fkf1 and ztl are reported to exhibit high rates of protein turnover, which facilitates their recycling to measure the day–night transition (Kim et al. 2007, Sawa et al. 2007). A recent study, however, has shown that the LOV domain of ztl undergoes adduct decay on a time scale of hours (Pudasaini and Zoltowski 2013). These findings therefore suggest that fkf1 and ztl possess distinct photochemical properties, which may contribute to their abilities to measure day-length accurately.

Although no structural information is available for ztl, fkf1 and lkp2, it is well established that these proteins function to control biological timing in Arabidopsis by regulating the stabilization of key regulatory targets. The expression of core circadian genes is controlled by factors such as the transcriptional repressor Timing of *CAB* expression 1 (TOC1) (Gendron et al. 2012, Huang et al. 2012, Pokhilko et al. 2012). Several studies indicate that the degradation of TOC1 is mediated by ztl and this process is inhibited by blue light (Mas et al. 2003, Kiba et al. 2007, Fujiwara et al. 2008). Light-mediated binding of the flowering- and circadian clock-associated protein Gigantea (GI) to ztl restricts its SCF activity, causing TOC1 to accumulate (Sawa et al. 2007). The interactions between ztl and GI are mediated by the ztl LOV domain (Fig. 4C), which results in the reciprocal stabilization of these proteins (Kim et al. 2007, Kim et al. 2013). GI binding therefore appears to limit the action of SCF^ztl^ on its regulatory targets.

A similar LOV-based stabilization mechanism has been reported for fkf1 and GI (Sawa et al. 2007, Fornara et al. 2009). However, in contrast to ztl, GI binding to fkf1 appears to promote SCF^fkf1^ activity. The onset of flowering by the fkf1 regulatory pathway involves targeted degradation of key transcriptional repressors of *FT* and *CO* expression. Transcriptional regulation of *CO* ensures that *FT* transcription is activated under long days (Andres and Coupland 2012). The kelch repeats of fkf1 are known to interact with Cycling DOF factors (CDFs) that bind to the promoter of *CO* and repress its transcription (Imaizumi et al. 2005, Sawa et al. 2007, Fornara et al. 2009). *CO* and *FT* expression is promoted under long days by fkf1-mediated degradation of these repressors, including CDF1 (Fig. 4C). The light-driven increase in SCF^fkf1^ activity involves GI binding to the fkf1 LOV domain (Sawa et al. 2007). Thus, GI binding to fkf1 and ztl can produce different effects on their SCF action. In addition to stabilizing GI, fkf1 also interacts with and stabilizes CO protein via its LOV domain to promote flowering under long days by activating *FT* expression (Song et al. 2012). The action of fkf1 on CO function is therefore mediated at both the transcriptional and post-translational level. A detailed description of the molecular mechanisms associated with circadian clock regulation and the photoperiodic control of flowering and how these different light input pathways are integrated is beyond the scope of this review, but can be found in several recently published articles (Andres and Coupland 2012, Ito et al. 2012, Lau and Deng 2012, Suetsugu and Wada 2013).

## Conclusions and Future Work

Since the cloning of the *CRY1* locus >20 years ago (Ahmad and Cashmore 1993), a great deal of progress has been made in characterizing the molecular basis of blue light perception in Arabidopsis. Three different classes of photoreceptors have been identified, all of which utilize flavin as a light-absorbing chromophore. However, many questions remain unanswered with respect to their mode of activation and signaling, and whether these photoreceptors operate as dimers. In the case of cryptochrome, structural information on the C-terminus will provide a clearer understanding as to how this region is important for receptor signaling. More work is needed to distinguish why the PHR domain initiates some aspects of cryptochrome signaling (CIB1) while others involve binding to the CCT (COP1–SPA1). With respect to phototropin, the function of receptor ubiquitination has yet to be resolved, as has the role of LOV1 photoactivation and the significance of receptor re-localization from the plasma membrane. Whether the mode of activation of ztl family members involves LOV-driven conformational changes within their C-terminus, as proposed for phototropins, requires further investigation, as will how GI binding to ztl and fkf1 can elicit different effects on their SCF activity. Plants also contain a unique LOV-containing protein that is unrelated to the phototropins or members of the ztl family (Kasahara et al. 2010), yet the function of this flavoprotein still remains unknown. Answering these unresolved aspects of blue light perception in plants would undoubtedly yield exciting advances in the years to come.

## Funding

The UK Biotechnology and Biological Sciences Research Council [BB/J016047/1 to J.M.C and a PhD Studentship [BB/F016735/1] to L.B.].

## Disclosures

The authors have no conflicts of interest to declare.

## Abbreviations

ABCB19: ABC transporter B family member 19
bHLH: basic helix–loop–helix
BLUS1: Blue Light Signaling 1
CCT: cryptochrome C-terminus
CDF: Cycling DOF factor
CIB1: cry-interacting bHLH1
CO: Constans
COP1: Constitutive Photomorphogenic 1
CPD: cyclobutane pyrimidine dimer
cry: cryptochrome
DASH: *Drosophila*–Arabidopsis–Synechocystis–Human
FADH·: neutral FAD radical
fkf1: Flavin-binding kelch repeat F-box 1
FT: Flowering Locus T
FTIR: Fourier transform infrared
GI: Gigantea
HY5: Long Hhypocotyl 5
KD: kinase domain
lkp2: LOV kelch protein 2
LOV: light, oxygen or voltage sensing
MTHF: 5,10-methenyltetrahydrofolate
NPH3: Non-Phototropic Hypocotyl 3
PAS, Per-ARNT-Sim; phot: phototropin
PHR: photolyase homology region
PHY: phytochrome
PKS4: Phytochrome Kinase Substrate 4
SAXS: small angle X-ray scattering
SCF: Skp Cullin F-box
SPA1: Suppressor of phyA 1
TOC1: Timing of CAB expression 1
UVR8: UV Resistance locus 8
ztl: Zeitlupe
